# Periodontitis affects glucoregulatory hormones in severely obese individuals

**DOI:** 10.1038/s41366-018-0253-4

**Published:** 2018-11-19

**Authors:** Anna Solini, Jean Suvan, Eleonora Santini, Stefano Gennai, Marta Seghieri, Stefano Masi, Morena Petrini, Francesco D’Aiuto, Filippo Graziani

**Affiliations:** 1 Department of Surgical, Medical, Molecular and Critical Area Pathology, University of Pisa, Pisa, Italy; 2 Periodontology Unit, University College London Eastman Dental Institute, London, UK; 3 Department of Clinical and Experimental Medicine, University of Pisa, Pisa, Italy

**Keywords:** Risk factors, Medical research

## Abstract

**Objective:**

To evaluate the effect of periodontitis (PD) on glucoregulatory hormones in obesity, never explored so far, a cross-sectional study was conducted in 110 severely obese, non-diabetic individuals.

**Methods:**

We collected clinical periodontal parameters, including probing pocket depth (PPD), bleeding on probing (BOP), clinical attachment level (CAL). Insulin, glucagon, GLP-1 and GIP were measured after 3 days of standardized diet.

**Results:**

Forty-seven subjects had periodontitis (PD+) and 63 did not (PD−). PD+ showed 30.3% of gingival sites with PPD > 4 mm, 55.2% of BOP sites and a mean CAL loss of 4.1 mm. Compared with PD−, PD+ had higher glucagon (26.60 [25.22] vs 3.93 [7.50] ng/l, *p* < 0.0001) and GIP levels (10.56 [13.30] vs 6.43 [8.43] pmol/l, *p* < 0.001), while GLP-1 was reduced (11.78 [10.07] vs 23.34 [16.80] pmol/l, *p* < 0.0001). Insulin did not differ. In PD+, after adjustment for confounders, PPD was positively related to glucagon (*β* = 0.424, *p* = 0.002) and inversely to GLP-1 (*β* = −0.159, *p* = 0.044).

**Conclusions:**

We describe for the first time an impaired incretin axis coupled with a relative hyperglucagonemia in obese non-diabetic individuals with PD, that might contribute to deteriorate their glucose tolerance and partially explain the higher risk of diabetes observed in these patients.

## Introduction

A bidirectional relationship links metabolic disorders like type 2 diabetes (T2D) or obesity and periodontitis (PD) [1]. The common soil between these chronic conditions is likely to be systemic, low-grade inflammation: dental plaque releases bioactive products, which in turn stimulate host responses at the gingival and systemic level, promoting the release of inflammatory cytokines like IL-1β, IL-6, and TNFα [2, 3]. These mediators are implicated in insulin resistance, impaired β-cell function, endothelial dysfunction, driving diabetes onset and the accelerating atherosclerosis marking it. Incretin peptides, principally GLP-1 and GIP, regulate islet hormone secretion, glucose concentrations, appetite and body weight, and immune function. A significant downregulation of the incretin secretion is detected in subjects with T2D, and recent studies suggest important anti-inflammatory effects of these molecules [4]. Thus, incretins might represent a pathway potentially involved in the dysregulation of glucose metabolism in patients with PD and at high risk of T2D, and the gingival inflammation could potentially exert a detrimental action also on the incretin axis. Obesity, rather than T2D, might represent a good model to address such issue, for the relative preservation of incretin function and, on the other hand, the high prevalence of periodontal disease [5]. The present study has been designed to evaluate whether serum levels of glucoregulatory hormones and incretins might be influenced by periodontal condition in obese non-diabetic individuals.

## Research design and methods

### Subjects

One hundred and ten obese (age 48 ± 10 years, 65 females, BMI 44.6 ± 8.9 kg/m^2^) patients were sequentially enrolled over 2015–2016 among participants in screening programs for periodontal disease. All participants signed an informed consent. Inclusion criteria were age <60 years, BMI > 35 kg/m^2^, absence of T2D (excluded by an oral glucose tolerance test, OGTT, performed 1 week before the study), no current smoking habits, no treatment with immunosuppressors/antibiotics/anti-inflammatory drugs and absence of systemic inflammatory diseases. All patients underwent a periodontal-screening visit; in those resulted positive to the periodontal screening record [6], a complete periodontal examination was performed. After 3 days of standardized 600 kcal diet, blood samples were drawn to measure routine biochemistry, high sensitivity CRP and plasma levels of insulin, glucagon, GLP-1 and GIP.

### Periodontal clinical parameters

Standard periodontal clinical parameters were registered using a UNC 15-mm periodontal probe by a calibrated examiner as previously reported [7]. The examiner recorded full-mouth pocket probing depth (PPD) and recessions at six sites per tooth (excluding third molars). Bleeding on probing (BOP) was recorded as local bleeding present within 30 s upon probing. Clinical attachment level (CAL) was calculated as the sum of PPD and gingival recession. Periodontitis was defined on interproximal CAL loss and radiographic evidence of bone loss as previously reported [8]. Periodontitis was stratified according to the recommendations of the American Academy of Periodontology [9].

### Assays

Plasma insulin was measured by an electro-chemiluminescence assay on a COBAS e411 (Roche, Indianapolis, IN, USA). Plasma GLP-1, GIP, and glucagon were assessed using a Multiplex technique (Millipore Corp, Billerica, MA, USA and Mercodia AB, Uppsala, SW). Circulating levels of IL-6, IL-1β, and TNFα were assessed by commercial ELISA kits (R&D Systems, Minneapolis, MN, USA) following manufacturer’s instructions.

### Statistics

Data were reported as mean ± SD, or median [IQR]. Differences between groups were analysed using ANOVA or Wilcoxon rank sum test for non-normally distributed variables. Relationships between variables were assessed using Spearman’s correlation analysis and multiple linear regression analysis, including stepwise model. Statistics were performed using JMP®7.0; a *p* value ≤ 0.05 was considered significant.

## Results

Participants were grouped based on the presence (PD+, *n* = 47) or absence (PD−, *n* = 63) of periodontitis. Clinical characteristics of the study participants are shown in Table 1. The two groups were balanced for age and gender, while BMI was significantly higher in PD− than and PD+ individuals. Percentage of former smokers was similar between the groups. Nearly half the population in both groups had the phenotype of metabolic syndrome. Among biochemical variables, no differences emerged for fasting plasma glucose and lipid profile, whereas hsCRP resulted elevated in both groups, but no differences between groups were observed. PD+ individuals had a mean number of 26 teeth, 30.3% of gingival sites with PPD > 4 mm, a mean 55.2% of BOP sites and an average CAL loss of 4.1 mm, indicating a generalized level of tissue loss.

**Table 1:** Clinical characteristics and circulating cytokine levels of the study participants

	PD+ (*n* = 47)	PD− (*n* = 63)	*p* value
Age (years)	48 ± 7	48 ± 12	0.910
Sex (F/M)	28/19	37/26	0.950
SBP (mmHg)	130.6 ± 18.9	130.1 ± 16.1	0.896
DBP (mm)	85.9 ± 10.6	83.1 ± 11.0	0.245
BMI (kg/m^2^)	41.4 ± 8.7	47.1 ± 8.3	**0.001**
Waist (cm)	116.1 ± 10.3	132.1 ± 11.7	**0.001**
Fasting plasma glucose (mmol/l)	5.37 ± 1.29	5.09 ± 1.23	0.100
Fasting plasma insulin (pmol/l)	90.29 [49.27]	80.97 [91.33]	0.075
HOMA-IR	3.38 [2.41]	2.71 [3.58]	**0.05**
Total cholesterol (mmol/l)	5.29 ± 1.13	5.02 ± 0.96	0.182
Triglycerides (mmol/l)	1.64 ± 0.83	1.61 ± 0.80	0.850
HDL cholesterol (mmol/l)	1.21 ± 0.35	1.26 ± 0.36	0.473
Metabolic syndrome^a^ (%)	52	48	0.932
hs CPR (mg/l)	4.31 ± 3.80	3.67 ± 2.62	0.290
IL-6 (pg/ml)	4.45 [2.10]	1.99 [1.34]	**<** **0.0001**
IL-1β (pg/ml)	3.53 [3.32]	1.99 [1.88]	**0.002**
TNFα (pg/ml)	2.82 [2.03]	2.03 [1.27]	**0.003**

^a^According to the IDF Consensus Worldwide Definition of the Metabolic Syndrome (2005)

Data are mean ± SD, or median[IQR]

**Fig. 1:**
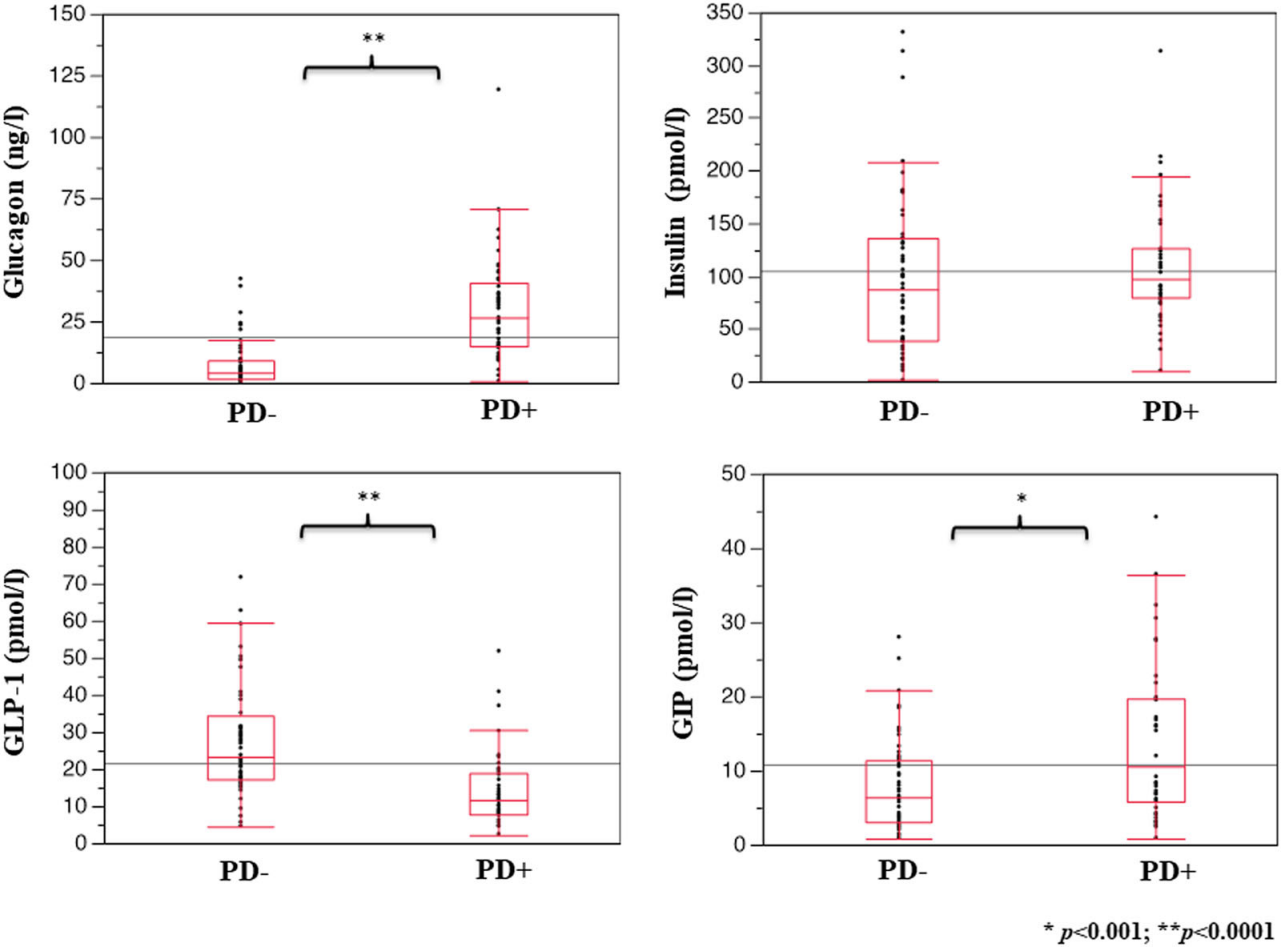
Plasma levels of glucagon, insulin, GLP-1, and GIP in obese non-diabetic individuals without (PD−) and with (PD+) chronic periodontitis. Data are reported as median and IQR

Glucoregulatory hormones are shown in Fig. 1. Plasma glucagon concentrations were six-fold higher in PD+ than in PD− subjects, while GLP-1 levels were markedly reduced. Plasma insulin did not differ between the groups, while GIP was increased in PD+ individuals.

In the PD+ group, CAL and PPD variables were linearly correlated with glucagon (*r* = 0.404, *p* = 0.003 and *r* = 0.456, *p* < 0.001, respectively). For PPD, such relationship still held at multiple regression analysis including BMI, age, and gender (*β* = 0.431, *p* = 0.002); moreover, when adding the incretin concentrations in the model, both glucagon (*β* = 0.424, *p* = 0.002) and GLP-1 (*β* = −0.159, *p* = 0.044) resulted associated (full model *r*^*2*^ = 0.30). After a stepwise approach results remained almost unchanged, with glucagon, GLP-1, and BMI explaining about the total variance of PPD. When comparing different stages of PD [9], glucagon levels were related with the severity of periodontitis (16.17 ± 9.95, 25.25 ± 4.34, 39.24 ± 4.45 ng/l from stages I to III; *p* = 0.03).

To better detect the relation between presence of periodontitis and systemic subclinical inflammation, we measured circulating levels of some inflammatory cytokines. As reported in Table 1, IL-6, IL-1β, and TNFα were significantly higher in the PD+ group. After modelling data by a multiple regression analysis, adjusting for sex, BMI, age and presence of PD, only IL-6 was significantly associated with the presence of PD (*β* = 0.88, *p* = 0.04).

## Conclusions

This study reports for the first time a strong impairment of incretin axis in obese individuals with severe periodontitis. An upregulation of glucagon, GLP-1, and GIP has been previously described in the gingival crevicular fluid of T2D subjects with periodontitis [10], while circulating levels of these hormones have never been related to the state of the gingivae. Our findings support the notion of a temporal association between periodontitis and obesity precedent to a derangement of glucose metabolism (normal OGTT and no difference in fasting glucose levels between the two groups), and eventually driving an increased risk of T2D in obese patients affected by periodontitis. This is further corroborated by the lower profiles of common risk factors of future T2D (lower BMI and a trend toward higher plasma glucose). However, our design does not allow inferring whether weight loss with consequent incretins’ variations may have a different impact in obese patients with or without periodontitis.

Intriguingly, CRP seems to mark a similar systemic low-grade inflammation in the two groups, suggesting a primary involvement of the mouth–gut axis, with a key role of the periodontitis and the oral inflammation as the main determinant of the observed hormone variations.

GLP-1 appears significantly reduced in obese patients with periodontitis. We have attempted to account for the common confounders like the type of consumed food, able to influence gut hormones (our study subjects followed the same diet for 3 days before the assessment). It is of interest that raised GLP-1 levels have been reported in critically ill individuals with a severe systemic inflammatory status [11] or after a major surgical stress [12]; here, diminished GLP-1 levels are observed in humans following a local and delimited, rather than a systemic, inflammatory status.

Looking more in detail to the hormone pattern coupled with periodontitis, glucagon levels were found to be elevated and correlated with the severity of the disease. Increased fasting glucagon concentrations may be responsible of the more pronounced insulin resistance [13], as observed in PD+ individuals according to HOMA-IR. The slightly higher fasting plasma glucose, likely due to marked increase in glucagon levels, in association with marginally raised insulin levels may account for the increased HOMA-IR in PD+ group. Moreover, this finding is reinforced by the fact that inflammatory cytokines levels, potentially associated with insulin resistance, are higher in PD+ group. In this view, the increased GIP levels deserve attention too, as they are able to stimulate glucagon secretion; such effect may help to stabilize glucose levels in healthy individuals, whilst in T2D and obese subjects these may contribute to glucose intolerance [14]. The hypothesis of a role of anatomical contiguity between mouth and gut in influencing this picture is reinforced by the lack of difference in insulin levels between obese with and without periodontal disease, according to previous data in carriers of metabolic syndrome [15]. Previous experimental works have shown that acute inflammation promotes GLP-1 secretion [11, 16]; in the present study, the chronic subclinical inflammation, associated with periodontitis, particularly IL-6, seems to be linked to an impaired incretin balance, this happens despite a lower BMI in PD+ individuals. A mere speculation on the mechanism behind the raised glucagon levels could be the trophic effect exerted by IL-6 on alpha-cells [17]; regarding GIP, beside its metabolic effects, it has been recently shown as it may promote adipose tissue inflammation and cytokine transcripts in humans [18]. In summary, this study shows for the first time a dysregulation of the incretin axis in obese individuals with periodontitis; we might speculate the potential role of the oral/gut microbiota in determining this clinical condition that might accelerate the deterioration of glucose homoeostasis frequently occurring in these individuals; a role for subclinical inflammation cannot be ruled out too. If confirmed in large further studies, our findings suggest that the incretin axis might represent a novel important target to prevent the development of T2D in patients with PD.
